# Ferroptosis-related differentially expressed genes serve as new biomarkers in ischemic stroke and identification of therapeutic drugs

**DOI:** 10.3389/fnut.2022.1010918

**Published:** 2022-11-10

**Authors:** Yinjiang Zhang, Yashuo Zhang, Rongfei Yao, Xu He, Linyi Zhao, Xiangyu Zuo, Binan Lu, Zongran Pang

**Affiliations:** ^1^School of Pharmacy, Minzu University of China, Beijing, China; ^2^Key Laboratory of Ethnomedicine, Minzu University of China, Ministry of Education, Beijing, China

**Keywords:** ferroptosis, ischemic stroke, biomarkers, immune microenvironment, subtypes

## Abstract

**Background:**

Iron is an essential nutrient element, and iron metabolism is related to many diseases. Ferroptosis is an iron-dependent form of regulated cell death associated with ischemic stroke (IS). Hence, this study intended to discover and validate the possible ferroptosis-related genes involved in IS.

**Materials and methods:**

GSE16561, GSE37587, and GSE58294 were retrieved from the GEO database. Using R software, we identified ferroptosis-related differentially expressed genes (DEGs) in IS. Protein-protein interactions (PPIs) and enrichment analyses were conducted. The ROC curve was plotted to explore the diagnostic significance of those identified genes. The consistent clustering method was used to classify the IS samples. The level of immune cell infiltration of different subtypes was evaluated by ssGSEA and CIBERSORT algorithm. Validation was conducted in the test sets GSE37587 and GSE58294.

**Results:**

Twenty-one ferroptosis-related DEGs were detected in IS vs. the normal controls. Enrichment analysis shows that the 21 DEGs are involved in monocarboxylic acid metabolism, iron ion response, and ferroptosis. Moreover, their expression levels were pertinent to the age and gender of IS patients. The ROC analysis demonstrated remarkable diagnostic values of LAMP2, TSC22D3, SLC38A1, and RPL8 for IS. Transcription factors and targeting miRNAs of the 21 DEGs were determined. Vandetanib, FERRIC CITRATE, etc., were confirmed as potential therapeutic drugs for IS. Using 11 hub genes, IS patients were categorized into C1 and C2 subtypes. The two subtypes significantly differed between immune cell infiltration, checkpoints, and HLA genes. The 272 DEGs were identified from two subtypes and their biological functions were explored. Verification was performed in the GSE37587 and GSE58294 datasets.

**Conclusion:**

Our findings indicate that ferroptosis plays a critical role in the diversity and complexity of the IS immune microenvironment.

## Introduction

There are genetic and environmental risk factors that interact to cause ischemic stroke (IS). Society and families are burdened by IS because it is the leading cause of disability (1). IS patients must continue taking medication for a long period after stroke onset, bringing about huge financial, mental, and time-wise burdens. IS risk factors include hypertension, diabetes, hyperlipidemia, and smoking. However, the molecular mechanism remains undetermined. Studies showed that early IS diagnosis can positively impact therapeutic outcomes and prognoses (2). Therefore, a better understanding of IS and identifying new biomarkers and therapeutic targets are urgently needed.

Iron is the most abundant trace element in the human body and is also considered indispensable for IS development (3). Ferroptosis is a unique type of programmed cell death distinguished by excessive iron buildup and lipid peroxidation (4). A recent study suggested that ferroptosis played an essential role in tumorigenesis and cancer progression (5). Additionally, ferroptosis is highly involved in many other diseases, such as IS and heart diseases (6). Moreover, research also proved that ferroptosis-related gene signatures could be used as a biomarker to diagnose, predict, and treat multiple diseases (7, 8). Nevertheless, the function of ferroptosis-related genes in IS is yet unclear.

In addition, stroke is often followed by post-stroke infection due to systemic immunosuppression, which has a worse outcome (9). It has been shown that immunomodulatory approaches, such as T-cell transfer and activators of natural killer T cells (NKTs), can reduce post-stroke immunosuppression (10). Immunomodulatory approaches can effectively manage stroke and its complications by targeting multiple elements of the immune system (11). Several well-known drugs, like azithromycin and metformin, can change the innate immune response. Both of these drugs are known to protect the brain after a stroke (12). So, it is important to figure out how immunosuppression works in stroke so that these drugs can be used to treat people. However, the immune mechanisms implicated in IS and IS-associated systemic immunosuppression are still poorly understood.

**FIGURE 1:**
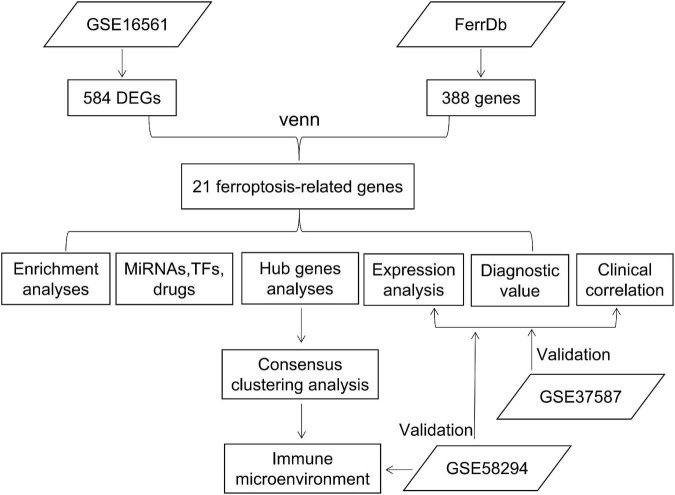
Workflow chart. DEGs, differentially expressed genes; TFs, transcriptional factors.

In this study, by comparing IS and normal samples in the GSE16561 dataset, differentially expressed genes (DEGs) were identified, which were then intersected with ferroptosis-related genes in the FerrDb database. We performed an enrichment analysis, as well as an investigation of expression levels and clinical significance. Unsupervised cluster analysis was performed on patients based on hub gene expression, and the characteristics of the immune microenvironment among different subtypes were analyzed. Validation was carried out in the GSE37587 and GSE58294 datasets (Figure 1).

## Materials and methods

### Data source

Data chips, microarrays, and gene expression data from GEO^1^ are available for research and analysis (13). Datasets were included and excluded according to the following criteria: (i) genomic wide expression mRNA microarray data had to be included, (ii) IS samples were required to be included, and (iii) specimen numbers must be greater than 30.

Three gene expression datasets, GSE16561 (14) and GSE37587 (15), derived from the GEO (GPL6883, Illumina HumanRef-8 v3.0 expression bead chip, array, Homo sapiens) were obtained with corresponding clinical data. GSE58294 (16) derived from the GEO (GPL570, Affymetrix Human Genome U133 Plus 2.0 Array, Homo sapiens) were obtained with corresponding clinical data. Data of the datasets were extracted from the total RNA of whole blood. Altogether, 39 IS and 24 normal whole blood samples were obtained from the GSE16561 cohort, 68 IS whole blood samples were obtained from the GSE37587 cohort, and 69 IS and 23 normal whole blood samples were obtained from the GSE16561 cohort.

A total of 388 ferroptosis-related genes were found in the FerrDb database^2^ (17; Supplementary Table 1) after removing duplicates. These genes include drivers, suppressors, and markers.

### Quality control

#### GSE16561

The raw expression profile GSE16561_RAW.tar was downloaded from the GEO database. Probes were annotated to their respective gene symbols *via* the GPL6883 platform file. Mean expression levels were used to compute gene symbols from several probes. Quantile normalization and log2 transformations were applied to raw data. Two abnormal IS samples (3100193_Stroke and 3100137_Stroke) were excluded based on principal component analysis (PCA). The following analysis included 37 IS and 24 normal samples. The GSE58294 dataset was also preprocessed according to the above process.

#### GSE37587

The raw expression profile GSE37587_non-normalized.txt.gz was downloaded from the GEO database. Probes were annotated to their corresponding gene symbols using the GPL6883 annotation file. Mean expression levels were used to compute gene symbols from several probes. Following the log2-transformation and quantile normalization, the raw expression files of the GSE16561 and the GSE37587 were combined. The batch correction was performed with the ComBat algorithm of the ‘‘sva’’ R package.^3^ The final dataset comprised 68 IS samples from GSE37587 and 24 normal samples from GSE16561. According to PCA, 3 abnormal IS samples were deleted (GSM922927, GSM922908, and GSM922905), thus 65 IS samples and 24 normal samples were included in the subsequent analysis. At the same time, a total of 107 IS samples from the two data sets were used for cluster analysis.

### Identification of ferroptosis-related differentially expressed genes and functional analysis

Limma^4^ (18) was used to identify DEGs between IS and normal samples in the GSE16561 (adjustment *p* < 0.05 and | log2FC| > 0.5). Heat maps were generated using the R package ‘‘pheatmap,’’^5^ exhibiting the top 20 genes with the most significant upregulation or downregulation, respectively. Twenty-one ferroptosis-related DEGs were obtained with a Venn diagram using the R package ‘‘Venn,’’^6^ and their expression correlation was calculated with the R package ‘‘corrplot’’^7^ and visualized using the ‘‘circlize’’ package^8^ (19). The ability of the 21 ferroptosis-related DEGs to distinguish between IS and normal samples was determined with PCA. Metascape (20) was used for functional analysis. Cut-off value: *P* < 0.05.

### Construct a diagnostic model of 21 ischemic stroke-associated ferroptosis genes

A diagnostic model was constructed by the least absolute shrinkage and selection operator (Lasso) analysis to analyze and identify the redundancy factors. Finally, receiver operating feature (ROC) scores were used to evaluate the diagnostic performance of the model.

### Bioinformatics analysis of 21 ferroptosis-related differentially expressed genes

In this study, Wilcoxon rank sum tests were used to examine the association between ferroptosis-related DEGs and age and gender of IS patients. *Via* the Enrichr platform^9^ (21), the transcription factors, upstream miRNAs, and small-molecular drugs of the 21 ferroptosis-related DEGs were predicted using the TRRUST, miRTarBase, and DSigDB databases, respectively. The ROC curve was generated using the ‘‘pROC’’ package^10^ (22) and visualized with the ‘‘ggplot2’’ package^11^ (23).

Based on the STRING database (24), PPI networks were constructed, in which the interactions with a score higher than 0.4 were considered statistically significant. The hub genes were selected with the plug-in CytoNCA (25) of the Cytoscape software V3.7.1 (26) and subjected to functional enrichment analysis using GeneMANIA^12^ (27). FDR < 0.05 was used as a cutoff point.

### Consensus clustering analysis

ConsensusClusterPlus (28) was used for cluster analysis. We used an agglomerative km clustering algorithm with one Pearson correlation distance and resampled 80% of the samples 10 times. Empirical cumulative distribution function plots were used to determine the optimal number of clusters.

### Immune cell infiltration analysis

Single sample gene set enrichment analysis (ssGSEA) was used to analyze the infiltration levels of immune cells based on 29 immune-related markers’ expression profiles. Also, CIBERSORT (29) was used to further analyze immune cell infiltration levels. Wilcoxon rank-sum tests were used to determine differences in immune cell proportions. The statistical significance threshold was set at *p* < 0.05.

### Gene set variation analysis

The GSVA (30) approach was used to examine important pathways and molecular processes by obtaining the h.all.v7.4.symbols.gmt and c2.cp.kegg.v7.4.symbols.gmt subsets from the Molecular Signatures Database (31). The minimum gene set was set to 5 and the maximum gene set was set to 5,000, and the enrichment scores were calculated for each sample in each gene set. The final enrichment score matrix was obtained. The differences in GSVA scores between subtypes for each gene set were compared using the limma package. FDR < 0.05 was used as a cutoff point.

### Identification of differentially expressed genes between different subtypes

Differentially expressed genes were screened for subtypes in the integrated dataset using the R package limma with | Fold Change| > 1.5 and FDR < 0.05. The differential genes were shown by volcano plot and heat map.

### Functional enrichment analysis

Biological functions were analyzed using the ClusterProfiler package (32), which includes GO and KEGG. Use the Benjamini–Hochberg method to adjust the *p*-value for multiple tests. *P* < 0.05 was used as a cutoff point.

### Statistical analysis

Statistical analysis was performed using R 4.1.0. Wilcoxon or Student’s *t*-test compared the two groups. Pearson’s or Spearman’s test determined the variables’ correlation. A Chi-square test was performed to compare two categorized variable groups. The statistical significance threshold was set at *p* < 0.05.

## Results

### Data preprocessing

#### GSE16561

**FIGURE 2:**
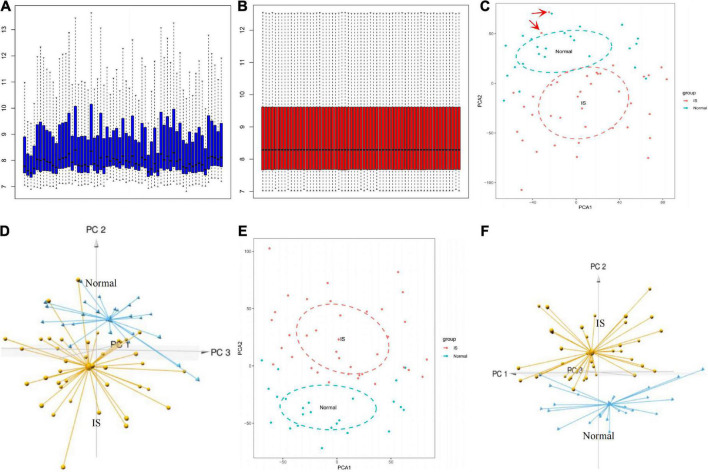
GSE16561 data set preprocessing. Box plot showing the gene expression level between different samples before **(A)** and after **(B)** normalization. 2D and 3D PCA plots demonstrated the distribution of samples before **(C,D)** and after **(E,F)** pretreatment. PCA, principal components analysis.

The box plot of the raw data demonstrated that gene expression levels were unevenly distributed across different samples (Figure 2A), which was processed *via* quantile normalization (Figure 2B). According to the 2D and 3D PCA plots (Figures 2C,D), two abnormal IS samples in the normal controls were deleted. Further validation distinguished between the groups and illustrated good clustering of samples within the same group (Figures 2E,F).

#### GSE37587 test set

Normalized gene expression data exhibited a uniform distribution in the samples (Supplementary Figure 1A). Three abnormal IS samples were excluded from the normal control based on the 2D and 3D PCA plots (Supplementary Figures 1B,C). PCA was repeated and demonstrated excellent discrimination between the groups and good clustering of samples within the same group (Supplementary Figures 1D,E).

### Ferroptosis-related differentially expressed genes differentiate ischemic stroke patients from normal controls

**FIGURE 3:**
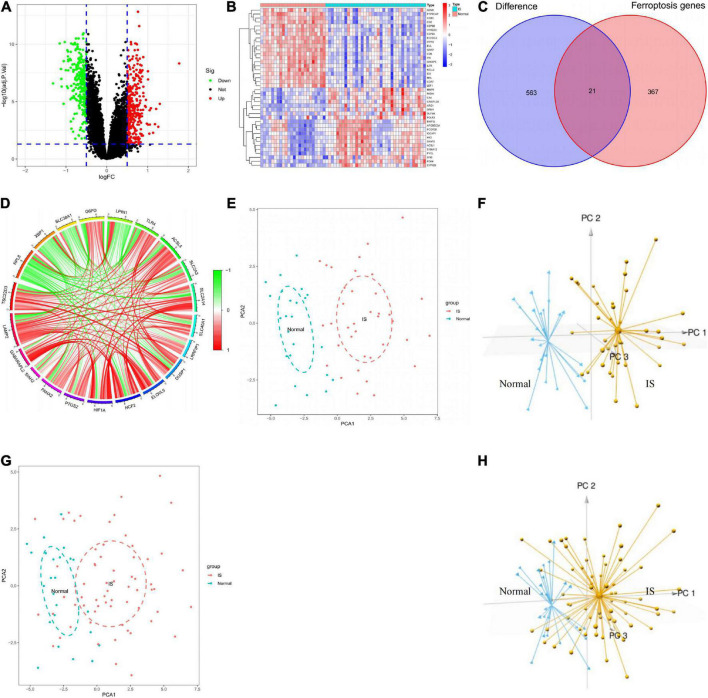
Analysis of differentially expressed genes. **(A)** Screening of DEGs shown by volcano plot. **(B)** Heatmap of the 40 genes expressed differently in IS samples compared to normal samples. **(C)** Venn diagram showing 21 ferroptosis-related DEGs. **(D)** Circle diagram showing the correlation of 21 ferroptosis-related DEGs. **(E,F)** 2D and 3D PCA plots showing PCA analysis based on 21 ferroptosis-related genes in GSE16561. **(G,H)** 2D and 3D PCA plots showing PCA analysis based on 21 ferroptosis-related genes in GSE37587. DEGs, differentially expressed genes; IS, ischemic stroke; PCA, principal components analysis.

According to | log2FC | > 0.5 and adjustment *p* < 0.05, 584 DEGs were obtained from the GSE16561 dataset, including 319 genes up-regulated and 265 genes down-regulated in IS samples (Figure 3A; Supplementary Table 2). The top 20 genes with the most significant upregulation or downregulation were selected to plot the Heat map (Figure 3B). To investigate the association between IS and ferroptosis, 21 intersecting genes were obtained between 584 DEGs and 388 ferroptosis-related genes (Figure 3C). Correlation analysis suggested correlations between the expression of those genes in the GSE16561 dataset (Figure 3D). Prominently, the 21 genes fully differentiated IS cases from normal controls in the GSE16561 dataset as analyzed by PCA (Figures 3E,F), which was verified in the test set GSE37587 (Figures 3G,H). Taken together, the 21 ferroptosis-related genes were highly heterogeneous between normal and IS tissues, and their expression changes may play a vital role in the initiation and development of IS.

### Enrichment analysis

**FIGURE 4:**
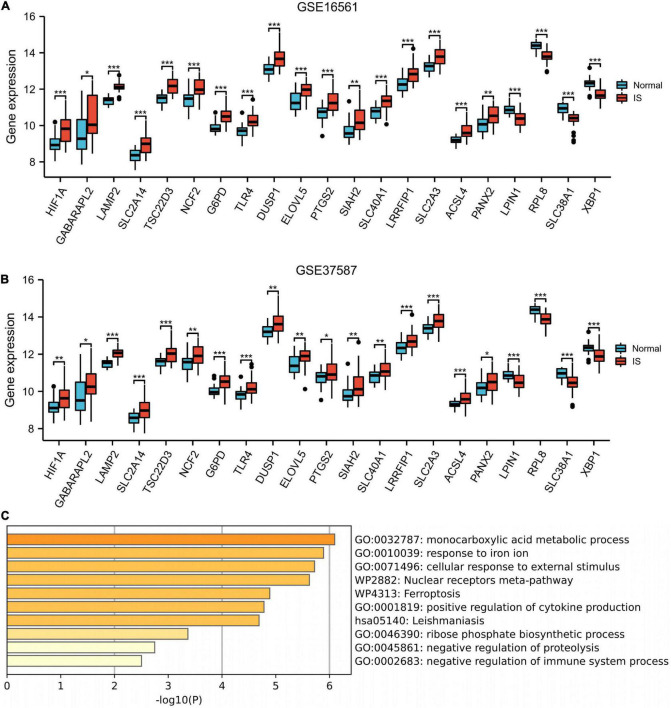
Enrichment analysis. Box plot described the expression pattern of the 21 ferroptosis-related genes between IS and normal samples in GSE16561 **(A)** and GSE37587 **(B)**. **(C)** 21 ferroptosis-related genes enrichment analysis. IS, ischemic stroke. *p < 0.05, **p < 0.01, and ***p < 0.001.

The box plot described the expression pattern of the 21 ferroptosis-related DEGs in IS and normal samples. In the two datasets, most of the 21 genes exhibited upregulation in IS tissues vs. normal tissues, except for LPIN1, RPL8, SLC38A1, and XBP1, which showed down-regulated expression (Figures 4A,B). Among 21 genes examined by enrichment analysis, monocarboxylic acid metabolism, iron ion responses, and ferroptosis were primarily enriched (Figure 4C).

### Clinical correlation analysis

**FIGURE 5:**
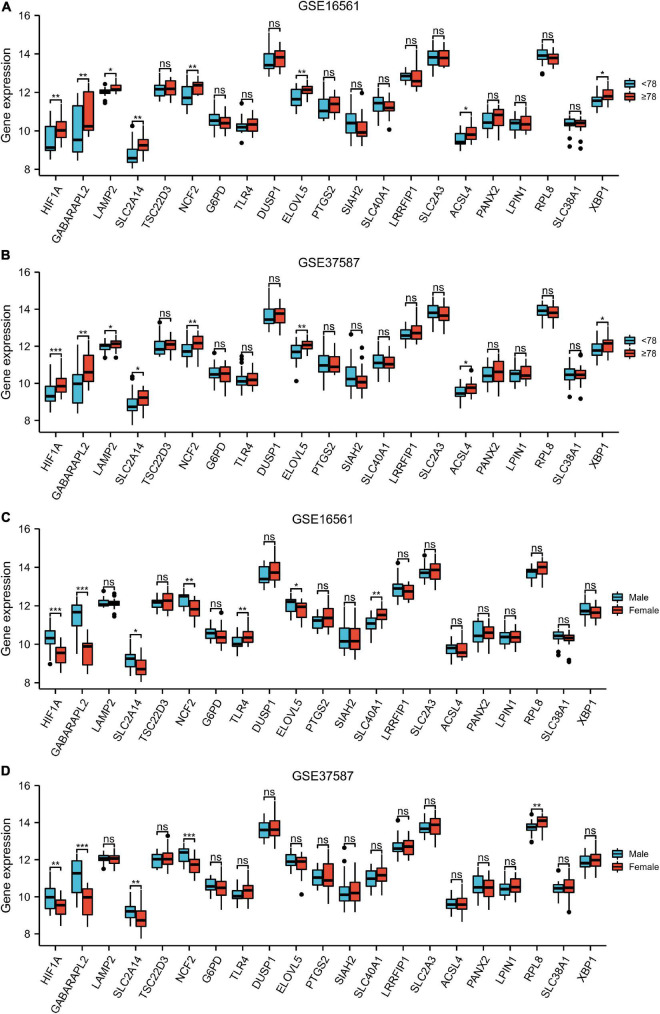
Clinical correlation analysis. Box plot showing the expression pattern of the 21 ferroptosis-related genes between <78 and ≥78 patients in GSE16561 **(A)** and GSE37587 **(B)**. Box plot showing the expression pattern of the 21 ferroptosis-related genes between male and female patients in GSE16561 **(C)** and GSE37587 **(D)**. ns: *p* ≥ 0.05, *p < 0.05, **p < 0.01, and ***p < 0.001.

The expression levels of HIF1A, GABARAPL2, LAMP2, SLC2A14, NCF2, ELOVL5, ACSL4, and XBP1 in patients ≥78 years old were significantly higher than those in patients < 78 years old in both the GSE16561 and GSE37587 datasets (Figures 5A,B). In the GSE16561 cohort, as compared to female patients, male patients displayed remarkably higher expression of HIF1A, GABARAPL2, SLC2A1, NCF2, and ELOVL5, while lower expression of TLR4 and SLC40A1 (Figure 5C). In the test set GSE37587, higher expression of HIF1A, GABARAPL2, SLC2A14, and NCF2 was also observed in male patients vs. female patients (Figure 5D). Therefore, the expression of these ferroptosis-related genes was interrelated with the age and gender of IS patients.

### Receiver operating feature analysis

**FIGURE 6:**
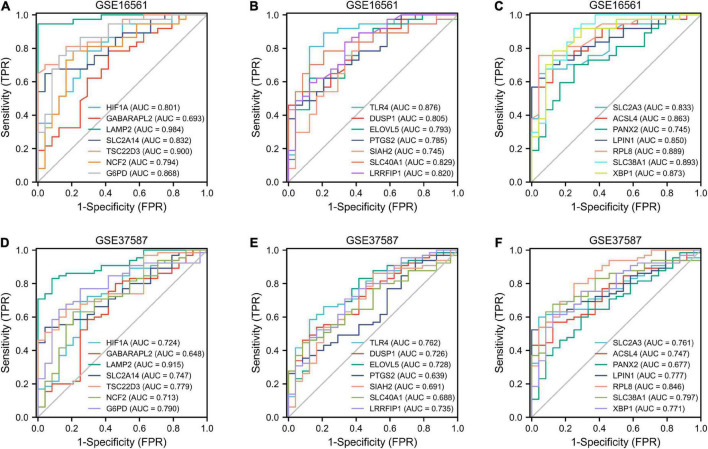
ROC analysis. Diagnostic ROC analysis of 21 ferroptosis-related genes in GSE16561 **(A–C)** and GSE37587 **(D–F)**. ROC, receiver operating characteristic; AUC, area under curve; FPR, false positive rate; TPR, true positive rate.

The ROC curves of the GSE16561 dataset revealed excellent accuracy of LAMP2 (AUC = 0.98), TSC22D3 (AUC = 0.90), SLC38A1 (AUC = 0.89), and RPL8 (AUC = 0.89) in distinguishing between the outcomes of normal and IS groups (Figures 6A–C). The ROC curves of the test set GSE37587 manifested moderate accuracy of LAMP2 (AUC = 0.92), RPL8 (AUC = 0.85), SLC38A1 (AUC = 0.80), and TSC22D3 (AUC = 0.78) in terms of differentiating between the outcomes of normal and IS groups (Figures 6D–F). In the GSE16561 dataset, based on lasso regression, we constructed a model composed of seven genes (LAMP2, LPIN1, TLR4, SLC2A3, LRRFIP1, PANX2, and GABARAPL2) to distinguish healthy subjects from IS patients (Supplementary Figures 2A,B). The AUC of GSE16561 was 1.000 in the training set, and the values of GSE37587 and GSE58294 AUC in the verification set were 0.961 and 0.730, respectively (Supplementary Figures 2C–E). Overall, these ferroptosis-related genes had remarkable diagnostic significance for IS patients.

### Transcript factor, upstream miRNA, and drug prediction

The transcription factors, upstream miRNAs, and related drugs of the 21 ferroptosis-related genes were predicted *via* the Enrichr platform. STAT6, IRF8, and HMGA1 were the main transcription factors retrieved from the TRRUST database (Table 1). Hsa-miR-548ag, hsa-miR-329-5p and hsa-miR-625-3p were the major upstream miRNAs according to the miRTarBase database (Table 2). Vandetanib, FERRIC CITRATE, etc., were the primary drugs predicted from the DSigDB database (Table 3). The identified transcription factors and miRNAs might be of prominent importance in the development of IS, and the predicted drugs can serve as potential drugs for IS.

**TABLE 1:** Transcriptional factor targets of 21 ferroptosis-related genes in IS.

Term	*P-value*	Odds ratio	Combined score
IRF8	5.74E-05	233.5672515	2280.803907
HMGA1	1.41E-04	140.0982456	1241.768431
STAT6	6.97E-06	100.7373737	1196.206221
CREM	3.10E-04	91.33180778	737.7313505
PGR	3.10E-04	91.33180778	737.7313505
ATF2	5.11E-04	69.99649123	530.5117162
USF2	0.001103204	46.62923977	317.5235245
CEBPB	0.001791576	36.15426497	228.6634283
SPI1	0.001911633	34.94561404	218.7524601
EGR1	0.003806749	24.34883721	135.6468757

IS, ischemic stroke.

**TABLE 2:** MicroRNA targets of 21 ferroptosis-related genes in IS.

Term	*P-value*	Odds ratio	Combined score
hsa-miR-548ag	5.92E-05	47.40238095	461.4566536
hsa-miR-625-3p	7.60E-04	56.73399716	407.4703927
hsa-miR-329-5p	1.65E-04	33.13166667	288.5803973
hsa-miR-606	0.001973063	34.37100949	214.0684295
hsa-miR-548ba	0.002429044	30.82198142	185.5562669
hsa-miR-548ai	2.50E-03	30.37376049	182.0091651
hsa-miR-570-5p	2.57E-03	29.93834586	178.5755424
hsa-miR-8076	0.003081354	27.20710868	157.3220116
hsa-miR-4798-3p	2.59E-02	41.57291667	151.8276652
hsa-miR-4499	3.32E-03	26.18289474	149.4851799

IS, ischemic stroke.

**TABLE 3:** Drug targets of 21 ferroptosis-related genes in IS.

Term	*P-value*	Odds ratio	Combined score
Vandetanib CTD 00004046	4.60E-08	151.4079696	2557.873945
FERRIC CITRATE CTD 00001186	6.89E-05	210.2	2014.429518
Flufenamic acid-(benzoic ring-13C6) TTD 00008058	9.48E-05	175.1491228	1622.485755
p-Phenylenediamine CTD 00001400	2.74E-07	93.78352941	1416.922104
CHLOROBENZENE CTD 00001495	1.25E-04	150.112782	1349.226508
Gossypol PC3 UP	3.67E-07	86.81917211	1286.427251
Tributyltin CTD 00000610	6.39E-06	103.890625	1242.619131
Dequalinium chloride HL60 DOWN	5.14E-07	79.44167498	1150.427383
Oligomycin CTD 00006434	1.59E-04	131.3355263	1148.713528
Mephentermine HL60 UP	7.87E-07	70.99108734	997.7694472

IS, ischemic stroke.

### Hub gene analysis

**FIGURE 7:**
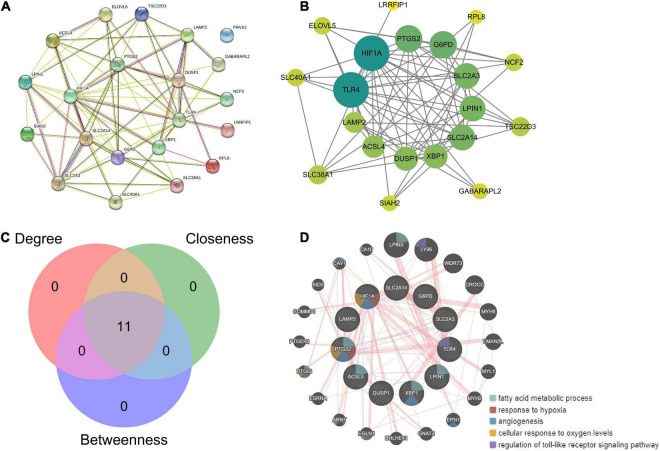
Hub gene analysis. **(A)** Based on the 21 ferroptosis-related genes, a PPI network was generated based on the STRING online database. **(B)** Hub genes were analyzed using the plug-in cytoNCA. **(C)** The top 11 hub genes were identified by calculating the betweenness, closeness, and degree. **(D)** Enrichment analysis of 11 hub genes by GeneMANIA online database.

Based on the 21 ferroptosis-related genes, a PPI network was generated based on the STRING online database (Figure 7A). Hub genes were analyzed using the plug-in cytoNCA. The top 11 hub genes were identified by calculating the Betweenness, Closeness, and Degree (Figures 7B,C). Based on enrichment analysis, the top 11 hub genes were highly enriched in fatty acid metabolism, hypoxia response, and angiogenesis (Figure 7D).

### Clustering analysis based on hub genes

**FIGURE 8:**
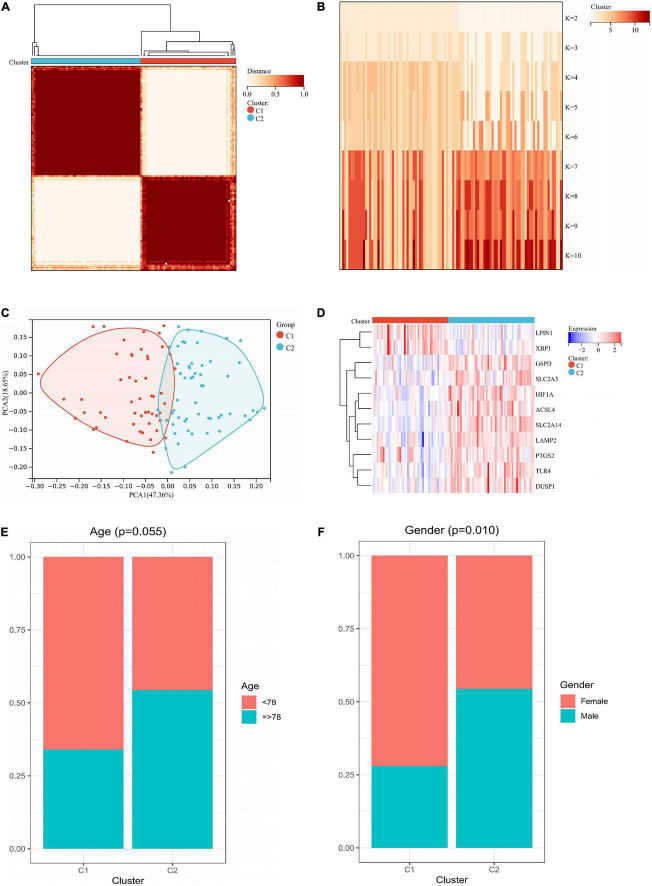
Unsupervised clustering of 11 hub genes. **(A)** Consensus matrix heatmap when *k* = 2. **(B)** Tracking plot showing the sample classification when *k* = 2–10. **(C)** PCA plots showing a remarkable difference in transcriptome between two subtypes. **(D)** Heatmap showing the expression of 11 hub genes in two subtypes. **(E)** Age ratio distribution in the two subtypes. **(F)** Gender ratio distribution in the two subtypes. PCA, principal components analysis.

We performed an unsupervised consistency clustering analysis on IS samples based on 11 hub genes (Supplementary Figures 3A–C). According to the average consistency evaluation within the cluster group, we choose the number of clusters as *K* = 2 (Figures 8A,B). We named these two subtypes C1 and C2, respectively. PCA analysis revealed significant differences between subtypes (Figure 8C). There was significant heterogeneity in the expression of 11 hub genes between subtypes (Figure 8D), and there was some association between subtypes and the age and sex of IS patients (Figures 8E,F).

### Characteristics of immune microenvironment in different subtypes

**FIGURE 9:**
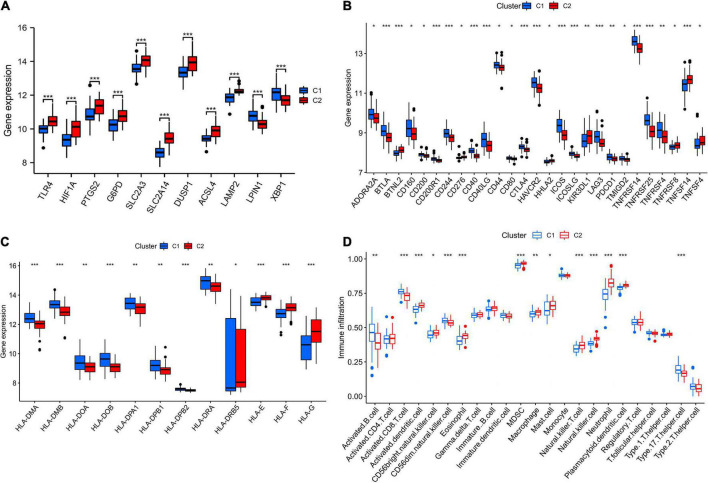
The features of the immunological microenvironment differ between subtypes. Box plots showing that there were differences in hub genes **(A)**, immune checkpoints **(B)**, HLA genes **(C)**, and immune cell infiltration **(D)** between the two subtypes. *p < 0.05, **p < 0.01, and ***p < 0.001.

Figure 9A showed that most of the hub genes were expressed at higher levels in C1 subtype than in C2. Most of the immune checkpoints and HLA genes were significantly upregulated in C1 compared to C2 subtype (Figures 9B,C). The C2 subtype was more immunoactive (p53 pathway, complement, IL6-JAK-STAT3 signaling, TNFA signaling *via* NFKB, chemokine signaling pathway, etc.) than the C1 subtype, as shown in Supplementary Figure 4. Based on the results of the ssGSEA algorithm, most immune cell infiltration levels differed significantly between C1 and C2 subtypes (Figure 9D). And the CIBERSORT algorithm analysis revealed a significant difference between C1 and C2 subtypes in terms of T cell infiltration (Supplementary Figures 5A,B).

### Functional enrichment analysis between different subtypes

**FIGURE 10:**
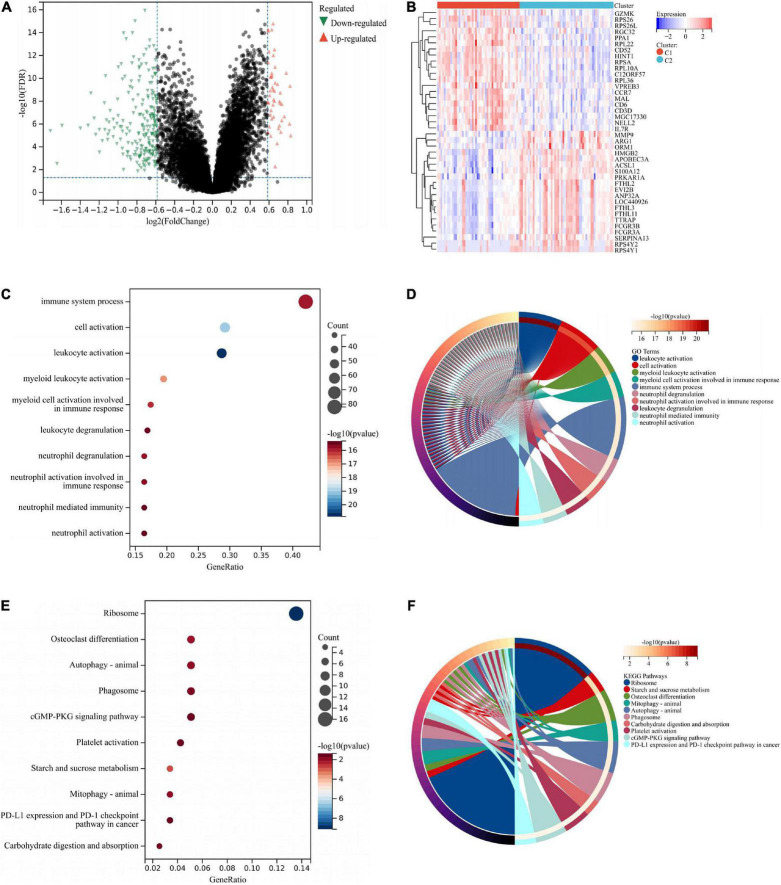
Functional analysis between two different subtypes. The DEGs were shown by volcano plot **(A)** and heat map **(B)** between two subtypes. **(C)** GO enrichment analysis was performed on the DEGs. GO terms are represented on the y-axis, gene ratios are shown on the x-axis, circle sizes refer to gene numbers, and colors represent *p*-values. **(D)** GO enrichment analysis of the DEGs. Different colors represent various significant GO terms and related enriched genes. **(E)** KEGG pathway analysis was performed on the DEGs. The y-axis represents different pathways, gene ratios enriched in relative pathways by the x-axis, circles represent gene numbers, and colors represent *p*-values. **(F)** KEGG pathway analysis of the DEGs. Different colors represent various significant pathways and related enriched genes. DEGs, differentially expressed genes; KEGG, Kyoto Encyclopedia of Genes and Genomes; GO, Gene Ontology.

Two hundred seventy-two DEGs were obtained from Cluster 1 and Cluster 2, of which 49 DEGs were upregulated in Cluster 1 and 223 DEGs were downregulated in Cluster 1 (Figure 10A). According to the heat map, two molecular subtypes could be distinguished by these DEGs (Figure 10B). Afterward, we analyzed DEGs among the two subtypes. As illustrated in Figures 10C,D, these DEGs were mainly associated with immune responses (immune system process, cell activation, leukocyte activation, etc.). Based on KEGG enrichment analysis, these DEGs were mainly associated with ribosomes, osteoclast differentiation, and autophagy (Figures 10E,F).

### External dataset validation

At the same time, we use GSE58294 to verify our analysis results and get similar results. First, we normalized the GSE58294 data set (Supplementary Figures 6A–C). Most 21 genes exhibited upregulation in IS tissues vs. normal tissues (Supplementary Figure 6D). The ROC curves also revealed excellent accuracy of LAMP2 (AUC = 0.94), TSC22D3 (AUC = 0.80), and SLC38A1 (AUC = 0.82) in distinguishing between the outcomes of normal and IS groups (Supplementary Figures 6E–G). We performed an unsupervised consistency clustering analysis on IS samples based on 11 hub genes and divided IS samples into two subtypes, C1 and C2 (Supplementary Figure 7A). Most of the immune checkpoints and HLA genes were significantly upregulated in C1 compared to C2 subtype (Supplementary Figures 7B,C). Most immune cell infiltration levels differed significantly between C1 and C2 subtypes (Supplementary Figure 7D). The C2 subtype was more immunoactive (complement, B cell receptor signaling pathway, VEGF signaling pathway, chemokine signaling pathway, etc.) than the C1 subtype (Supplementary Figure 7E).

## Discussion

Ferroptosis is a unique type of programmed cell death involved in metabolism, redox biology, and various diseases (33), such as degenerative disorders, carcinogenesis, stroke, and traumatic brain injury. A recent study demonstrated that ferroptosis is critical for the progress of cerebral stroke (34). By understanding the association between ferroptosis and IS, new biomarkers and approaches to diagnosis and treatment can be developed.

In the present study, using GSE16561 dataset, 21 ferroptosis-related DEGs were identified. IS-related pathways like monocarboxylic acid metabolism, iron ion response, and ferroptosis were enriched. Monocarboxylic acids such as lactic acid (35), pyruvate (36), and ketone body (37) are closely related to IS. ACSL4 was reported to be a potential therapeutic target for IS, as it could aggravate IS by promoting ferroptosis (38). Therefore, the 21 ferroptosis-related DEGs identified in this study may contribute significantly to IS through these pathways.

Furthermore, clinical correlation analysis indicated that the expression of DEGs was related to patients’ age and gender. It has been long recognized that stroke incidence is higher in men than in women globally (39). Moreover, men have a higher age-adjusted incidence of stroke than women (40).

Further analysis revealed that LAMP2, RPL8, and SLC38A exhibited excellent diagnostic performance for IS patients in both the GSE16561 and GSE37587 datasets. Tao et al. (41) revealed that miR-207 mediated the ischemic injury and spontaneous recovery by participating in the lysosome pathway *via* regulating LAMP2. After intracerebral hemorrhage, human brain RPL8 mRNA expression increased, suggesting it may be a therapeutic target (42). To conclude, the 21 ferroptosis-related DEGs might be critical to IS.

The transcription factors, upstream miRNA, and drugs that correspond to the 21 ferroptosis-related DEGs were also confirmed in this study. The transcription factors identified mainly were STAT6, IRF8, and HMGA1. STAT6/Arg1 promoted microglia/macrophage efferocytosis and inflammation resolution in stroke mice (43); IRF8 protected against cerebral ischemic-reperfusion injury (44); has-miR-196a alleviated ischemic brain injury in mice by directly targeting HMGA1 (45). The three miRNAs identified were hsa-miR-548ag, hsa-miR-329-5p, and hsa-miR-625-3p. The expression of hsa-miR-625-3p was correlated with cholesterol levels (46) and hsa-miR-625-3p was reported to be interrelated with cerebral infarction (47). Given these findings, the identified transcription factors and miRNAs were essential to the ferroptosis dysfunction in IS. Among the drugs, vandetanib could be used to treat thyroid and non-small cell lung cancer (48), and it might serve as a potential drug for IS.

Protein-protein interaction analysis identified 11 hub genes out of the 21 ferroptosis-related genes, which were majorly enriched in fatty acid metabolic process, response to hypoxia, and angiogenesis. Research showed that the fatty acid metabolic process was closely correlated with stroke (49). Hypoxia could induce IS (50), while angiogenesis-associated factors could act as biomarkers for IS patients (51). Therefore, the 11 hub genes are presumably of vital importance in IS.

In this study, using consistent clustering, we identified two subtypes (C1 and C2) in IS samples based on 11 ferroptosis-related genes. C1 contained 50 samples, and C2 contained 57 samples. Significant heterogeneity between the two subgroups was confirmed by immunoassay and enrichment analysis. At the same time, we use GSE58294 to verify our analysis results and get similar results. According to many studies, ferroptosis plays a vital role in immunity (52, 53). It is thought that ferroptotic cells activate innate immunity and release pro-inflammatory factors in various diseases, attracting many different immune cells to the area (54). In IS, BBB breaks down, allowing immune cells to flood into the central nervous system. Our findings suggested that NK and mast cells infiltrated less in C1 than in C2. Kong et al. (55) reported that the number of NK cells was reduced in IS patients. The mast cells contributed to the development of IS by speeding up BBB disruption and magnifying neuroinflammation by releasing cytokines (56). Two limitations in this study warrant mention. The ferroptosis-related DEGs with significance in IS might not be comprehensively included. Moreover, validations are required in further *in vivo* and *in vitro* experiments.

## Conclusion

The current study identified 21 ferroptosis-related DEGs in IS, which were pertinent to the age and gender of IS patients and had an excellent diagnostic performance. Vandetanib, FERRIC CITRATE, etc., were identified as potential drugs for IS. In addition, we proposed a molecular classification based on ferroptosis-related genes, namely C1 and C2 subtypes in IS. In conclusion, our findings may help to design immunotherapies for IS patients.

## Data availability statement

The datasets presented in this study can be found in online repositories. The names of the repository/repositories and accession number(s) can be found in the article/Supplementary material.

## Author contributions

YJZ, BL, and ZP designed the study, performed data analysis, and wrote the manuscript. YSZ, RY, XH, LZ, and XZ gathered clinical and expression data. All authors contributed to the article and approved the submitted version.

## Footnotes

1
http://www.ncbi.nlm.nih.gov/geo

2
http://www.zhounan.org/ferrdb/current/

3
https://bioconductor.org/packages/sva/

4
https://bioconductor.org/packages/limma/

5
https://CRAN.R-project.org/package=pheatmap

6
https://CRAN.R-project.org/package=venn

7
https://CRAN.R-project.org/package=corrplot

8
https://CRAN.R-project.org/package=circlize

9
http://amp.pharm.mssm.edu/Enrichr/

10
https://CRAN.R-project.org/package=pROC

11
https://CRAN.R-project.org/package=ggplot2

12
http://genemania.org/

## Supplementary material

Supplementary Figure 1 GSE37587 data set preprocessing. **(A)** Box plots showing gene expression levels between different samples after normalization. 2D and 3D PCA plots demonstrated the distribution of samples before **(B,C)** and after **(D,E)** pretreatment. PCA, principal components analysis.

Supplementary Figure 2 Construct a diagnostic model of 21 IS-associated ferroptosis genes. **(A)** Least absolute shrinkage and selection operator (LASSO) coefficient profiles of 21 IS-related ferroptosis genes. **(B)** Plots of the 10-fold cross-validation error rates. **(C–E)** The model’s discrimination ability for healthy and IS samples was analyzed by ROC curve and evaluated by AUC value. IS, ischemic stroke; ROC, receiver operating characteristic; AUC, area under curve; FPR, false positive rate; TPR, true positive rate.

Supplementary Figure 3 Cluster parameter analysis. **(A)** Cumulative distribution curve when *k* = 2–10. **(B)** Relative alterations in the area under CDF curve. **(C)** Sample clustering consistency when *k* = 2–10.

Supplementary Figure 4 Two subtypes differ in biological function. Heatmap showing the enrichment levels of Hallmark **(A)** and KEGG **(B)** gene sets in two subtypes.

Supplementary Figure 5 CIBERSORT to assess immune cell infiltration. **(A)** Bar plot showing the proportion of 22 immunocytes in two subtypes. **(B)** Violin plot showing the ratio of immune cells between two subtypes.

Supplementary Figure 6 The expression level and diagnostic value of 21 IS-associated ferroptosis genes were verified in the GSE58294 data set. **(A)** Box plot showing the gene expression level between different samples after normalization. **(B,C)** 2D and 3D PCA plots demonstrated the distribution of samples after pretreatment. **(D)** Box plot described the expression pattern of the 21 ferroptosis-related genes between IS and normal samples. **(E–G)** Diagnostic ROC analysis of 21 ferroptosis-related genes in GSE58294. PCA, principal components analysis; IS, ischemic stroke; ROC, receiver operating characteristic; AUC, area under curve; FPR, false positive rate; TPR, true positive rate. Ns: *p* ≥ 0.05, **p* < 0.05, ***p* < 0.01, and ****p* < 0.001.

Supplementary Figure 7 The unsupervised cluster analysis of 11 hub genes was verified in the GSE58294 data set. **(A)** Based on 11 hub gene expression levels, IS samples were divided into two subtypes, C1 and C2. Box plots showing that there were differences in immune checkpoints **(B)**, HLA genes **(C)**, and immune cell infiltration **(D)** between the two subtypes. **(E)** Heatmap showing the enrichment levels of KEGG gene sets in two subtypes. KEGG, Kyoto Encyclopedia of Genes and Genomes. **p* < 0.05, ***p* < 0.01, and ****p* < 0.001.
