# Supplementary figures and images for: Ferroptosis-related differentially expressed genes serve as new biomarkers in ischemic stroke and identification of therapeutic drugs
Source: Front Nutr. 2022 Nov 10;9:1010918. doi: 10.3389/fnut.2022.1010918 (PMC9686348; doi:10.3389/fnut.2022.1010918)

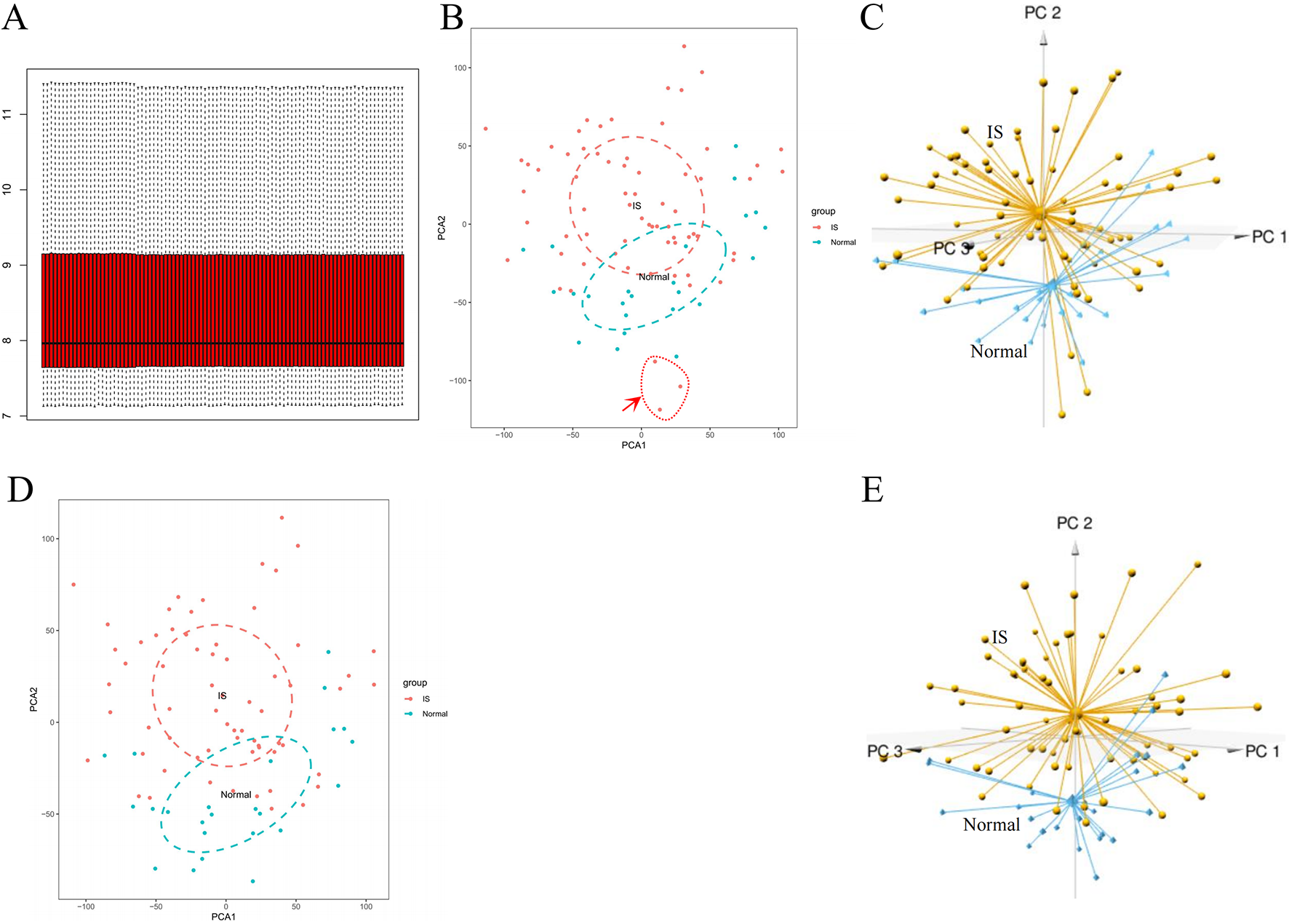

Supplement: Supplementary Figure 1 — GSE37587 data set preprocessing. (A) Box plots showing gene expression levels between different samples after normalization. 2D and 3D PCA plots demonstrated the distribution of samples before (B,C) and after (D,E) pretreatment. PCA, principal components analysis. [file Image_1.TIF]

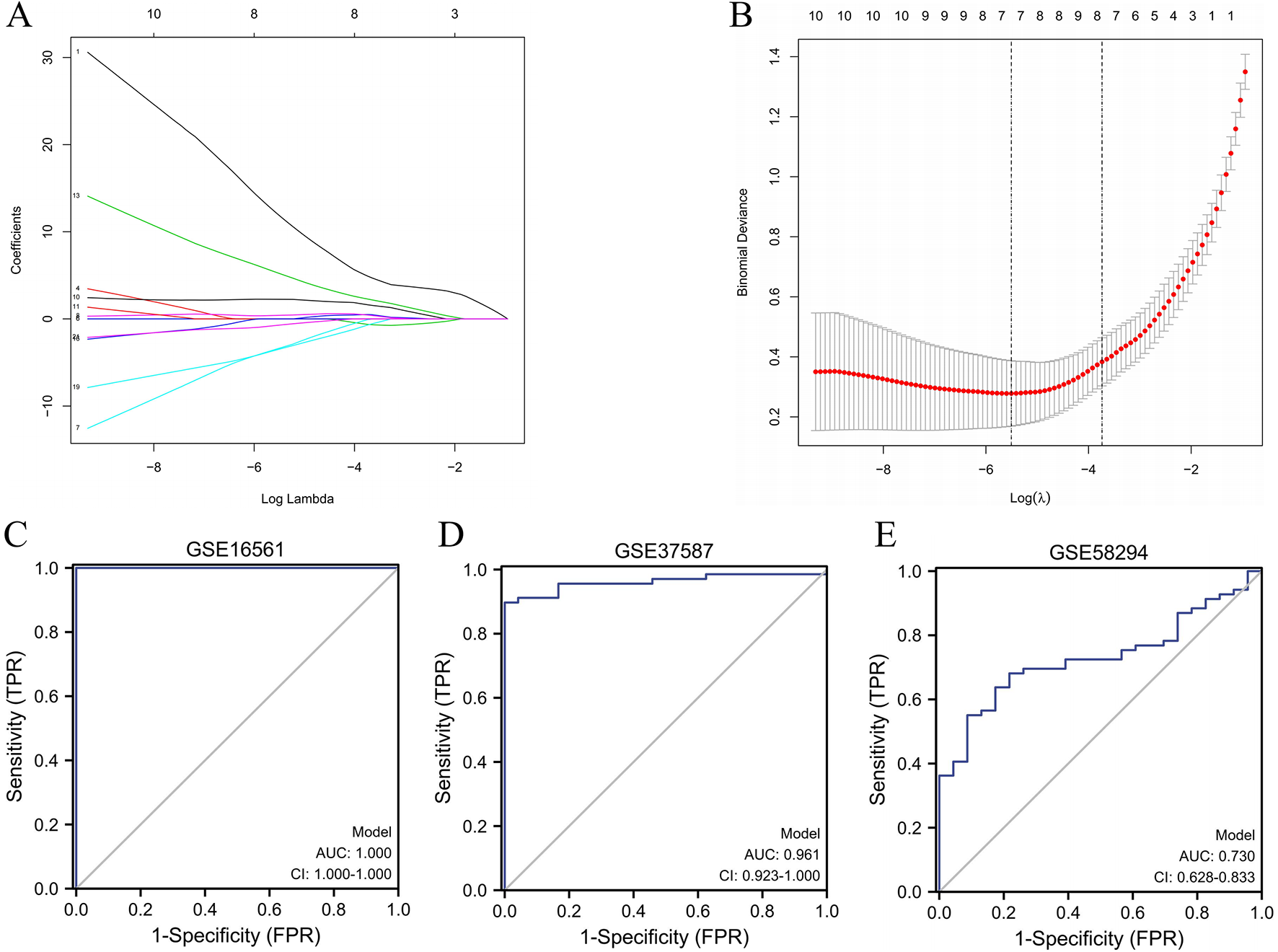

Supplement: Supplementary Figure 2 — Construct a diagnostic model of 21 IS-associated ferroptosis genes. (A) Least absolute shrinkage and selection operator (LASSO) coefficient profiles of 21 IS-related ferroptosis genes. (B) Plots of the 10-fold cross-validation error rates. (C–E) The model’s discrimination ability for healthy and IS samples was analyzed by ROC curve and evaluated by AUC value. IS, ischemic stroke; ROC, receiver operating characteristic; AUC, area under curve; FPR, false positive rate; TPR, true positive rate. [file Image_2.TIF]

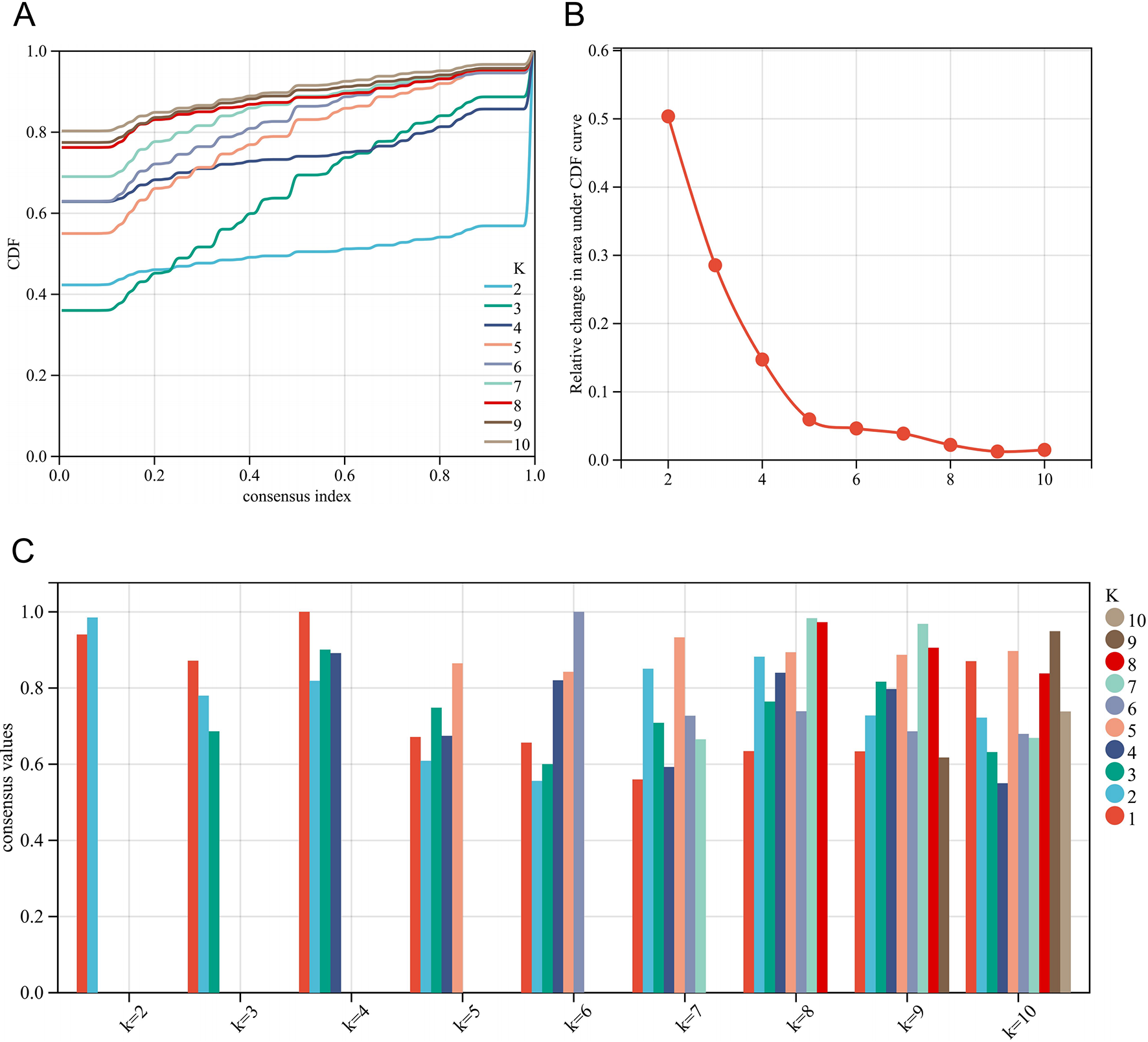

Supplement: Supplementary Figure 3 — Cluster parameter analysis. (A) Cumulative distribution curve when k = 2–10. (B) Relative alterations in the area under CDF curve. (C) Sample clustering consistency when k = 2–10. [file Image_3.TIF]

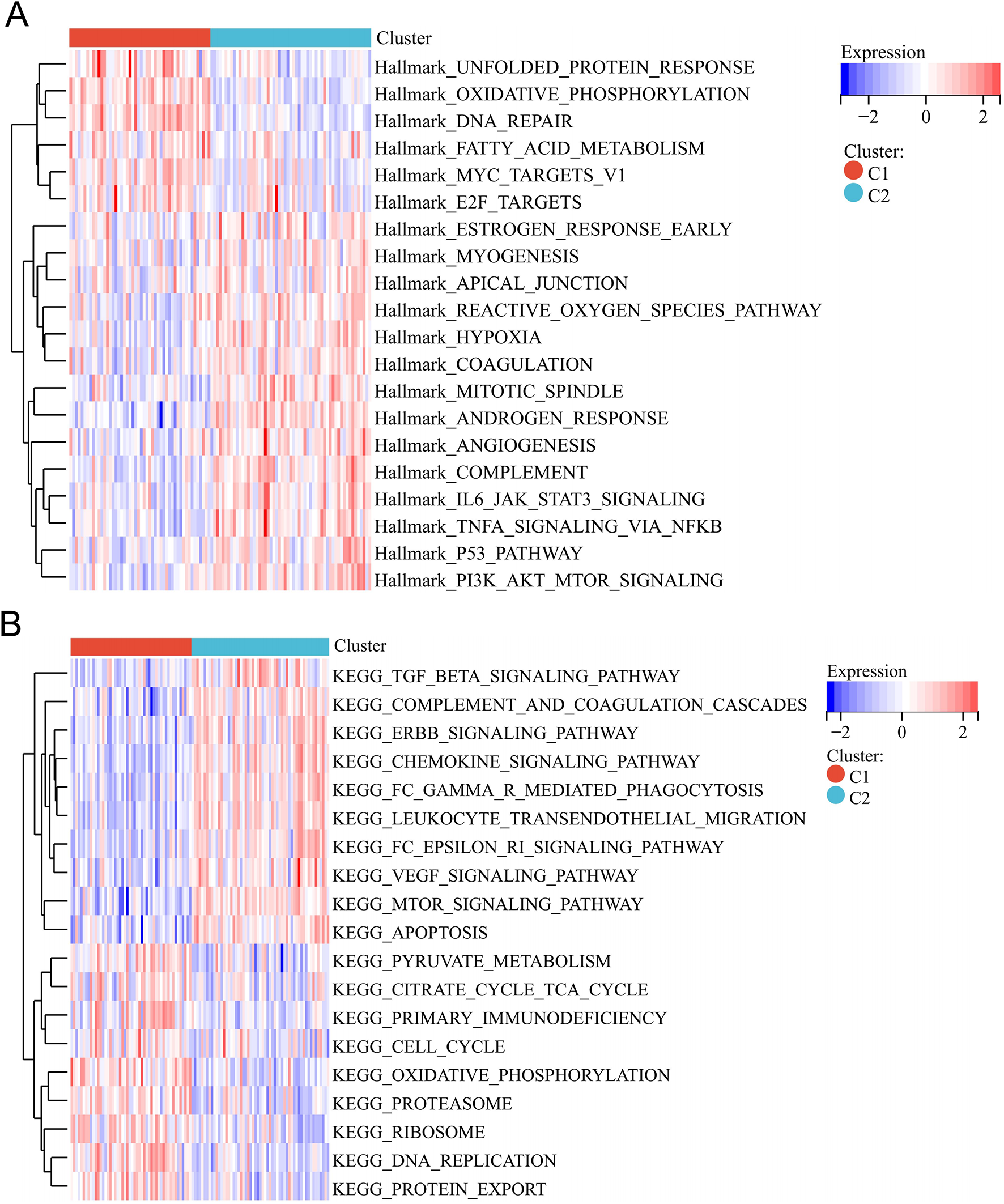

Supplement: Supplementary Figure 4 — Two subtypes differ in biological function. Heatmap showing the enrichment levels of Hallmark (A) and KEGG (B) gene sets in two subtypes. [file Image_4.TIF]

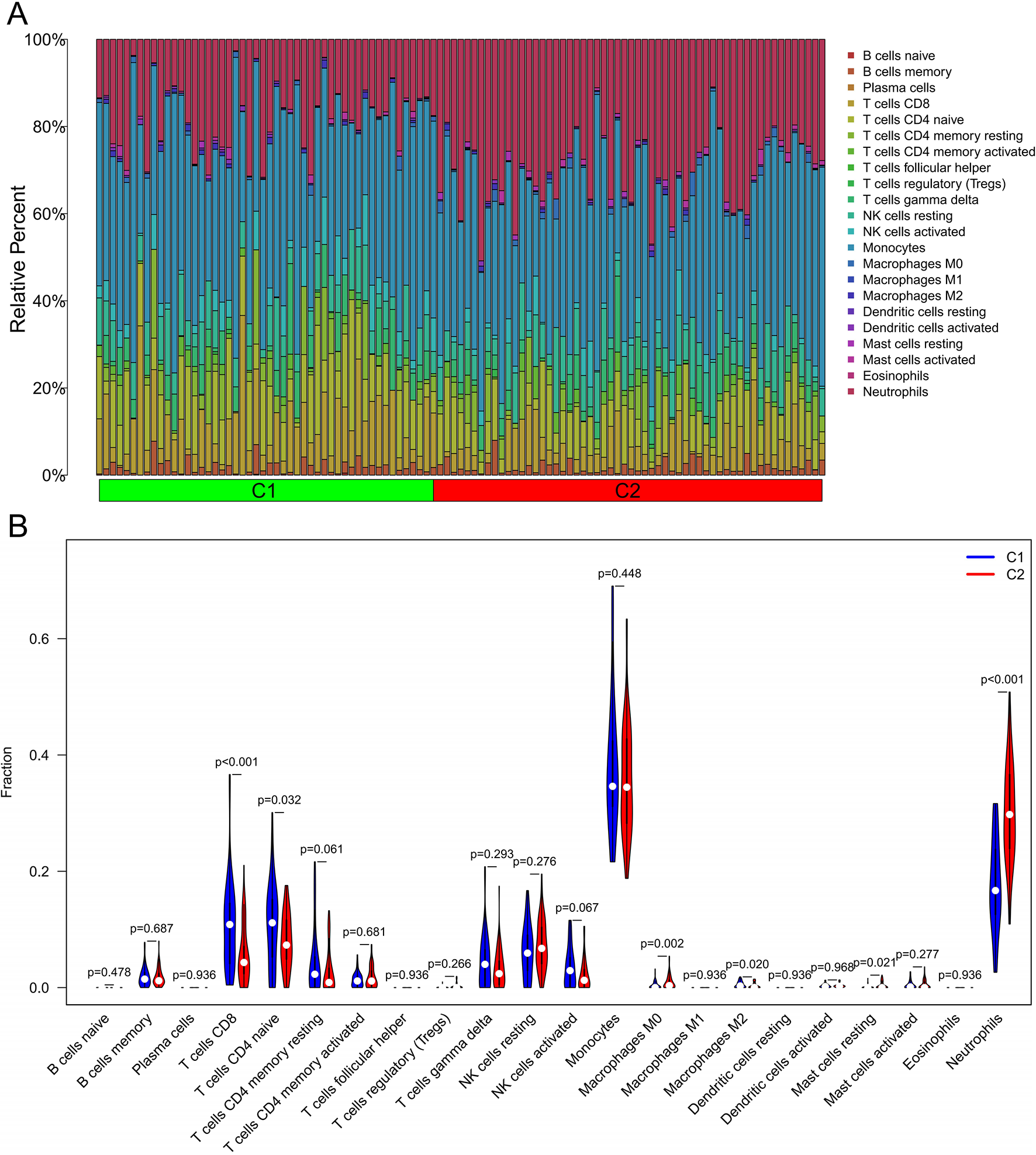

Supplement: Supplementary Figure 5 — CIBERSORT to assess immune cell infiltration. (A) Bar plot showing the proportion of 22 immunocytes in two subtypes. (B) Violin plot showing the ratio of immune cells between two subtypes. [file Image_5.TIF]

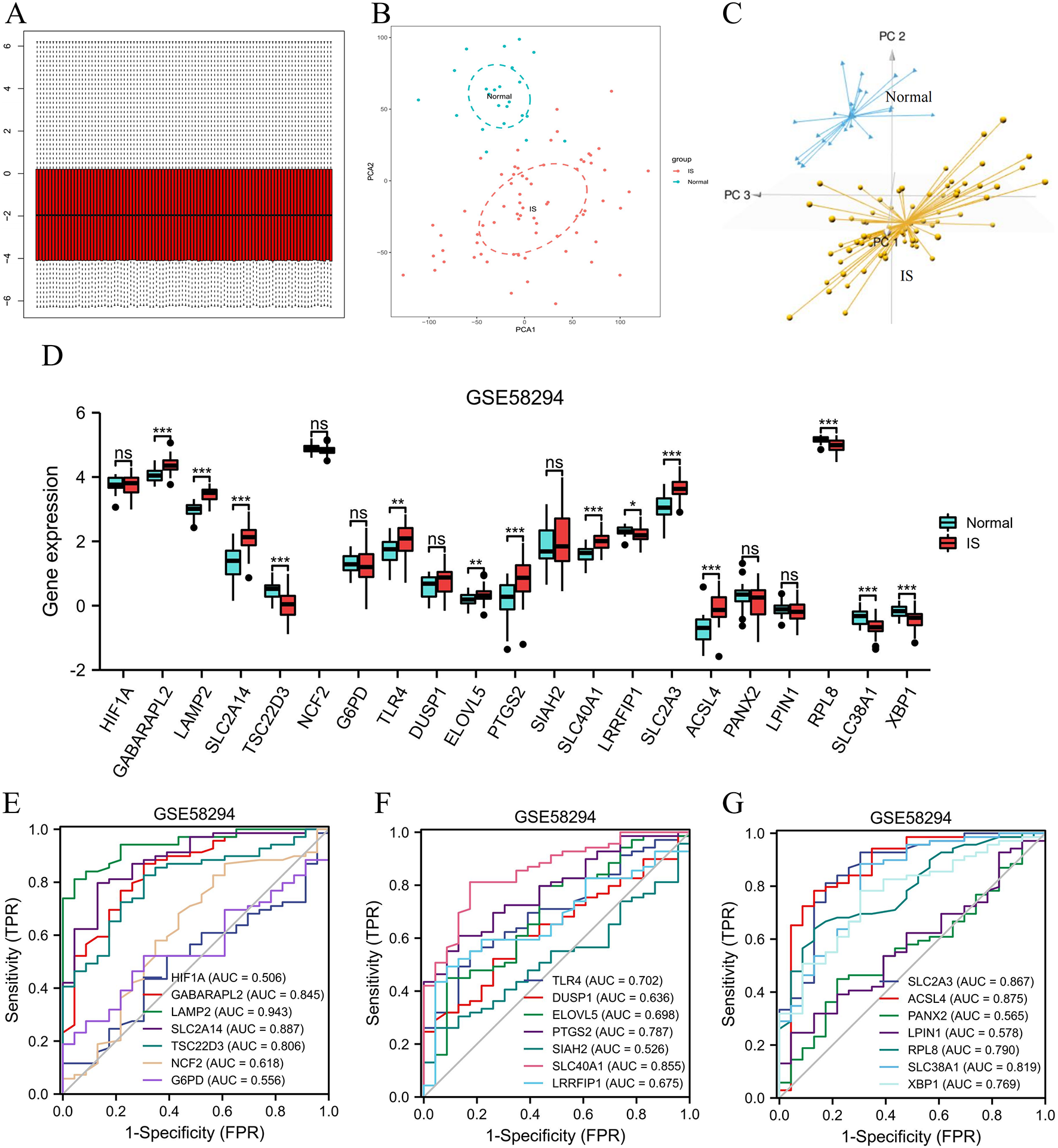

Supplement: Supplementary Figure 6 — The expression level and diagnostic value of 21 IS-associated ferroptosis genes were verified in the GSE58294 data set. (A) Box plot showing the gene expression level between different samples after normalization. (B,C) 2D and 3D PCA plots demonstrated the distribution of samples after pretreatment. (D) Box plot described the expression pattern of the 21 ferroptosis-related genes between IS and normal samples. (E–G) Diagnostic ROC analysis of 21 ferroptosis-related genes in GSE58294. PCA, principal components analysis; IS, ischemic stroke; ROC, receiver operating characteristic; AUC, area under curve; FPR, false positive rate; TPR, true positive rate. Ns: p ≥ 0.05, *p < 0.05, **p < 0.01, and ***p < 0.001. [file Image_6.TIF]

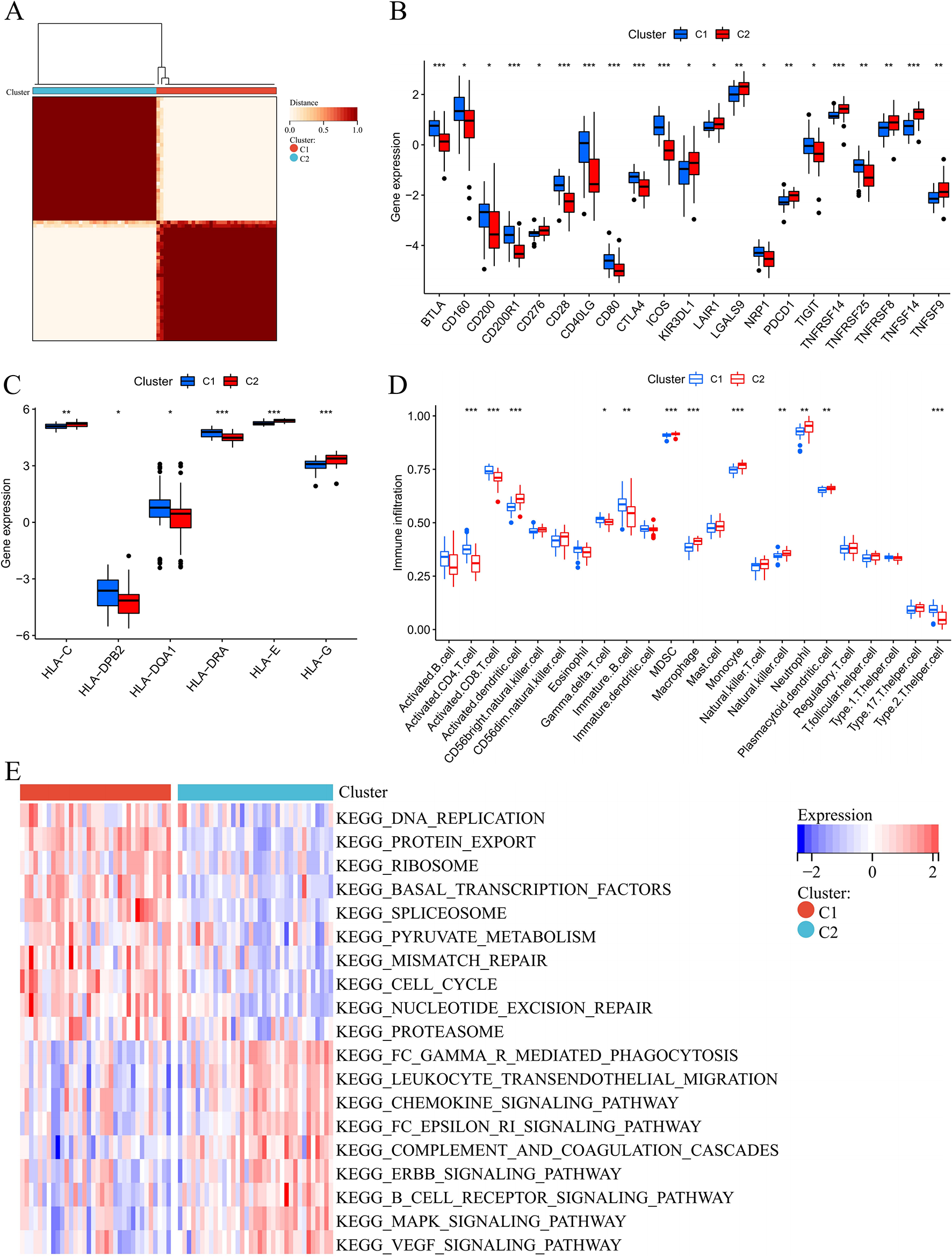

Supplement: Supplementary Figure 7 — The unsupervised cluster analysis of 11 hub genes was verified in the GSE58294 data set. (A) Based on 11 hub gene expression levels, IS samples were divided into two subtypes, C1 and C2. Box plots showing that there were differences in immune checkpoints (B), HLA genes (C), and immune cell infiltration (D) between the two subtypes. (E) Heatmap showing the enrichment levels of KEGG gene sets in two subtypes. KEGG, Kyoto Encyclopedia of Genes and Genomes. *p < 0.05, **p < 0.01, and ***p < 0.001. [file Image_7.TIF]
